# Physiology

**Published:** 1876-08

**Authors:** 


### III. PHYSIOLOGY.

1. *A Theory of the Dicrotic Pulse.* F. P. HENRY, M. D. (*Phila. Med Times*, April 29, 1876.)

This theory is believed to be novel. It is held that "the pulse is composed of two elements," the *wave* of blood and the corresponding wave of dilatation of the artery, and "the blood-*current* itself, which travels so much slower than the undulation that it arrives at the wrist during the recoil of the artery. It is the blood current which causes the reascent in the line of the sphygmographic tracing." "The undulation precedes the current;" "it consists mainly of an up-and-down movement of the particles, that is, one at right angles to the current, which movement is intermittent. The current consists of a continuous onward movement of the blood-stream, whose volume is increased at each ventricular contraction." The current moves more slowly than the wave; so that while the latter may precede the former at the wrist by one-half second, or about half a pulsation, at the inner malleolus the current may be so far behind the wave with which it left the heart that its impulse against the walls of the artery may be exactly synonomous with the impulse of the wave produced by the systole following, so that the two acting in unison, a more vigorous pulsation may be produced.

The theory of Morey, that the dicrotic pulse is due to the concussion "received by the blood at the point of division of the aorta into the two primitive iliacs," is denied, for the reason that there is not time for a wave produced by concussion at this point to travel backward and produce the phenomena of dicrotism.

